# Does canine inflammatory bowel disease influence gut microbial profile and host metabolism?

**DOI:** 10.1186/s12917-016-0736-2

**Published:** 2016-06-16

**Authors:** Jia Xu, Adronie Verbrugghe, Marta Lourenço, Geert P. J. Janssens, Daisy J. X. Liu, Tom Van de Wiele, Venessa Eeckhaut, Filip Van Immerseel, Isabel Van de Maele, Yufeng Niu, Guido Bosch, Greet Junius, Brigitte Wuyts, Myriam Hesta

**Affiliations:** Department of Nutrition, Genetics and Ethology, Faculty of Veterinary Medicine, Ghent University, Heidestraat 19, 9820 Merelbeke, Belgium; Present Address: Department of Clinical Studies, Ontario Veterinary College, University of Guelph, 50 Stone Road East, Guelph, N1G 2W1 ON Canada; Laboratory of Microbial Ecology and Technology (LabMET), Faculty of Bioscience Engineering, Ghent University, Coupure Links 653, 9000 Ghent, Belgium; Department of Pathology, Bacteriology and Avian Diseases, Faculty of Veterinary Medicine, Ghent University, Salisburylaan 133, 9820 Merelbeke, Belgium; Department of Medicine and Clinical Biology of Small Animals, Faculty of Veterinary Medicine, Ghent University, Salisburylaan 133, 9820 Merelbeke, Belgium; Laboratory of Aquaculture & Artemia Reference Center, Ghent University, Rozier 44, 9000 Ghent, Belgium; Animal Nutrition Group, Department of Animal Sciences, Wageningen University, De Elst 1, 6708 WD Wageningen, The Netherlands; Private Small Animal Clinic ‘Dierenartsencentrum Hond en Kat’, Emiel Clauslaan 134, 9800 Astene, Belgium; Laboratory of Metabolic Disorders, Ghent University Hospital, De Pintelaan 185, 9000 Ghent, Belgium

**Keywords:** Acylcarnitine profile, Butyrate-producing bacteria, Citrulline, Dog, Fermentation, Inflammatory bowel disease, *Lactobacillus*, Microbiota, Short-chain fatty acid

## Abstract

**Background:**

Inflammatory bowel disease (IBD) refers to a diverse group of chronic gastrointestinal diseases, and gut microbial dysbiosis has been proposed as a modulating factor in its pathogenesis. Several studies have investigated the gut microbial ecology of dogs with IBD but it is yet unclear if this microbial profile can alter the nutrient metabolism of the host. The aim of the present study was to characterize the faecal bacterial profile and functionality as well as to determine host metabolic changes in IBD dogs.

Twenty-three dogs diagnosed with IBD and ten healthy control dogs were included. Dogs with IBD were given a clinical score using the canine chronic enteropathy clinical activity index (CCECAI). Faecal short-chain fatty acids (SCFA) and ammonia concentrations were measured and quantitative PCR was performed. The concentration of plasma amino acids, acylcarnitines, serum folate, cobalamin, and indoxyl sulfate was determined.

**Results:**

No significant differences in the abundance of a selection of bacterial groups and fermentation metabolites were observed between the IBD and control groups. However, significant negative correlations were found between CCECAI and the faecal proportion of *Lactobacillus* as well as between CCECAI and total SCFA concentration. Serum folate and plasma citrulline were decreased and plasma valine was increased in IBD compared to control dogs. Increased plasma free carnitine and total acylcarnitines were observed in IBD compared with control dogs, whereas short-chain acylcarnitines (butyrylcarnitine + isobutyrylcarnitine and, methylmalonylcarnitine) to free carnitine ratios decreased. Dogs with IBD had a higher 3-hydroxyisovalerylcarnitine + isovalerylcarnitine to leucine ratio compared to control dogs.

**Conclusions:**

Canine IBD induced a wide range of changes in metabolic profile, especially for the plasma concentrations of short-chain acylcarnitines and amino acids, which could have evolved from tissue damage and alteration in host metabolism. In addition, dogs with more severe IBD were characterised by a decrease in faecal proportion of *Lactobacillus*.

**Electronic supplementary material:**

The online version of this article (doi:10.1186/s12917-016-0736-2) contains supplementary material, which is available to authorized users.

## Background

Canine idiopathic inflammatory bowel disease (IBD) is a group of disorders characterized by persistent or recurrent clinical signs of gastro-intestinal disease of undetermined cause associated with histological evidence of inflammation in the small or large intestinal mucosa [1]. It has been considered to be the most common cause of chronic diarrhoea and vomiting in dogs [2]. Gut microbiota play a critical role in host-microbial interactions and gut health. Gut dysbiosis, an unbalanced microbiota composition, is often seen in dogs with IBD and may contribute to the pathogenesis of this disease [3–7]. In humans, IBD has been associated with a decrease in butyrate-producing bacteria including the major butyrate producing bacteria, clostridial cluster IV and XIVa, *Faecalibacterium prausnitzii*, and the terminal gene to produce butyrate, butyryl-CoA acetate-CoA transferase (BCoAT) gene [8–10]. Similarly, a lower abundance of *Faecalibacterium* spp. has been reported in dogs with IBD [4, 7]. However, the relative proportion of *Faecalibacterium* is much lower in healthy dogs compared to humans [7, 11, 12]. Therefore, assessing the major butyrate producing clusters and the functional gene that expresses BCoAT gene could be important in exploring the role of butyrate producing bacteria in canine IBD.

The changes in the gut microbial populations in IBD are becoming clearer [3–7], however, the functional relationship between microbes and the host is less well understood in both humans and dogs. A major function of gut microbiota is degradation of non-digestible dietary residues, yielding fermentation end-products which can be beneficial or harmful to the host, such as short-chain fatty acids (SCFA) and putrefactive substances. Several studies have shown changes in fermentation metabolites in humans IBD patients [13–15]. However, no study has investigated the fermentation end products in dogs with IBD yet. Further, gut-derived SCFA are rapidly absorbed from the gut lumen and enter into systemic circulation at different concentration. Based on the concentrations in peripheral and portal blood, about 75 % of acetate, 90 % of propionate and 95 % of butyrate are taken up during a single pass of blood through the human liver [16]. The metabolism of SCFA requires activation of coenzyme (Co) A and intracellular CoA bound acylgroups are then transported from the cytoplasm to the mitochondria by carnitine groups [17]. Hence, short-chain acylcarnitines (i.e. acetylcarnitine, propionylcarnitine, butyrylcarnitine) are measures of respective SCFA-CoA and reflect the major pathways by which SCFA influence cellular metabolism [18].

Host metabolic differences have been reported in both animals and humans. Canine IBD was associated with increased serum abundance of 3-hydroxybutyrate, hexuronic acid, and gluconic acid lactone [3]. Similarly, an ulcerative colitis (UC) mice model showed an increase in serum ketone bodies, a drop in serum glucose as well as decreases in several amino acids (e.g. tyrosine, glutamine, and alanine) [19]. An altered amino acid profile was also observed in both UC and Crohn’s disease (CD) patients, with for instance, increased levels of isoleucine and decreased valine in serum [20]. Thus, IBD is characterised by significant changes in host metabolites in humans but data are still lacking for canine IBD.

Although several studies investigated the microbial ecology of IBD patients [3–7], it is yet unclear if the altered microbial profile also results in an altered nutrient metabolism of the host. The aim of the study was to evaluate faecal microbial profile and functionality especially focussing on butyrate-producing microbiota and the concomitant fermentation and host metabolic profile in dogs with IBD. We hypothesized that canine IBD is associated with changes in faecal microbial and metabolic profile. The findings of this study might provide potential interesting candidates for diagnosing IBD in both humans and dogs.

## Methods

### Animals

The European Directive 2010/63/EU and institutional guidelines of Belgian Council for Laboratory Animal Science for the care and use of animals were followed. The present study used opportunistic sampling in the IBD dogs that were presented in the clinic as no additional manipulations were needed besides the necessary procedure for normal diagnosis and treatment. For the control dogs, samples were collected after euthanasia for reasons not related to the study, thus, an approval from the Ethical Committee (of the Faculty of Veterinary Medicine, Ghent University, Belgium) was not required. Informed written consent was obtained from the owners of all IBD dogs participating in the study and all dogs received a high standard of veterinary care.

Twenty-three dogs, 15 admitted at the Small Animal Clinic of Ghent University (Belgium) and eight at a local private small animal clinic ‘Dierenartsencentrum Hond en Kat’, Astene (Belgium), diagnosed with IBD were included in the IBD group. The minimum diagnostic evaluation performed in all IBD dogs included complete blood counts, serum biochemistry, urinalysis, faecal parasitology, abdominal ultrasound, and histopathologic review of mucosal biopsy specimens. The inclusion criteria for the IBD group were adapted from World Small Animal Veterinary Association [2]: (1) chronic (> three weeks) persistent or recurrent gastrointestinal signs such as but not limited to vomiting and diarrhoea; (2) histopathologic evidence of mucosal inflammation; (3) inability to document other causes of gastrointestinal inflammation; (4) inadequate response to dietary, antibiotic and anthelmintic therapies alone. The requirement of the clinical response to anti-inflammatory or immunosuppressive agents was not included because anti-inflammatory drugs may influence gut microbiota [21]. Additionally, no dogs received antibiotics for at least three weeks before sampling.

Dogs with IBD were allocated a clinical score using the canine chronic enteropathy clinical activity index (CCECAI) [22]. Briefly, the CCECAI score is based on nine criteria, each scored on a scale from 0–3: attitude/activity, appetite, vomiting, stool consistency, stool frequency, weight loss, albumin levels, ascites and peripheral edema, pruritus. After summation, the total score is determined to be clinically insignificant (0–3), mild (4–5), moderate (6–8), severe (9–11), or very severe (≥12) IBD.

Ten healthy dogs, four from the Department of Medical Physiology, University Medical Centre Utrecht (The Netherlands) and six from an animal shelter (Wase Dierenbescherming, Sint-Niklaas, Belgium) were included in the control group. The control dogs were euthanized for research purposes unrelated to the present study or for behavioural problems. All dogs were deemed healthy based on physical exams, complete blood counts, and serum biochemistry and none of the dogs received antibiotic treatment for at least three weeks before sampling.

### Sample collection

At presentation in the clinic for IBD dogs, and immediately after euthanasia for control dogs, blood samples (5 mL, normally taken for general health check-up) were drawn from the jugular vein. Plasma and serum were obtained from all dogs, except for one IBD dog (due to improper sample storage) by centrifugation at 1620 *g* for 15 min at 4 °C and stored at – 20 °C until analysis. Upon diagnosis, faecal samples were collected from 15 dogs in the IBD group by natural voiding of faeces or by use of rectal swabs. For all dogs in the control group, faecal samples were obtained from the rectum immediately after euthanasia. All faecal samples were frozen in dry ice and stored at – 80 °C until analysis.

### Analytical methods

Serum cobalamin and folate concentrations were analyzed by a commercial laboratory (Algemeen Medisch Laboratorium, Antwerp, Belgium) using commercially available ARCHITECT B12 and ARCHITECT Folate assays on ARCHITECT *i* System (Abbott Diagnostics). Plasma amino acids and acylcarnitine profile was determined according to Zytkovicz et al. [23]. Faecal SCFA concentrations were determined via gas chromatography after extraction with diethyl ether [24]. Ammonia was analyzed by steam distillation and titration according to Bremner and Keeney [25].

### Bacterial DNA extraction

Bacterial DNA extraction of the faecal samples was performed according to Vanhoutte et al. [26]. DNA concentration was measured in triplicate using the Nanodrop ND 1000 spectrophotometer (NanoDrop Technologies).

### Quantitative PCR

DNA quantification by qPCR was performed using the C1000 Thermal Cycler (Bio-Rad, Hercules CA, USA). The amplification and detection were carried out in 96-well plates using SensiMixTM SYBR No-ROX Kit (Bioline Reagents Ltd, UK). Each reaction was done in triplicate in 12 μL total reaction mixture using 2 μL of 50 ng of the DNA sample. The primer sets used in this study are listed in Table 1. A melting curve analysis was done after amplification to confirm specificity of the reaction. The quantification was done using standard curves made from known concentrations of plasmid DNA containing the respective amplicon for each set of primers.

**Table 1:** Primer set used in the present study

Target	Primers (5’ → 3’)	References
Bacteria V3 region	PRBA338f ACTCCTACGGGAGGCAGCAG	[49]
	PRUN518r ATTACCGCGGCTGCTGG	
Total Bacteria	fwd CGGYCCAGACTCCTACGGG	[50]
	rev TTACCGCGGCTGCTGGCA	
Firmicutes	fwd GGAGYATGTGGTTTAATTCGAAGCA	[51]
	rev AGCTGACGACAACCATGCAC	
*Enterobacteriaceae*	fwd CATTGACGTTACCCGCAGAAGAAGC	[52]
	rev CTCTACGAGACTCAAGCTTGC	
Bacteriodetes	fwd GGARCATGTGGTTTAATTCGATGAT	[51]
	rev AGCTGACGACAACCATGCAG	
*Lactobacillus*	fwd GGAATCTTCCACAATGGACG	[53]
	rev CGCTTTACGCCCAATAAATCCGG	
Clostridial cluster I	fwd TACCHRAGGAGGAAGCCAC	[54]
	rev GTTCTTCCTAATCTCTACGCAT	
Clostridial cluster IV	fwd ATGCAAGTCGAGCGA(G/T)G	[55]
	rev TATGCGGTATTAATCT(C/T)CCTTT	
Clostridial cluster XIVa	fwd CGGTACCTGACTAAGAAG	[56]
	rev AGTTT(C/T)ATTCTTGCGAAC	
Butyryl-CoA acetate-CoA transferase	fwd AAGGATCTCGGIRTICAYWSIGARATG)	[57]
	rev GAGGTCGTCICKRAAITYIGGRTGNGC	
Dissimilative sulphate-reducing bacteria gene	fwd AACAACATHGARTTYATG	[58]
	rev TAGCAGTTACCRCARTACAT	

### Calculations

Total acylcarnitines were calculated as the sum of plasma concentration of acetylcarnitine (C2), propionylcarnitine (C3), butyrylcarnitine and isobutyrylcarnitine (C4), tiglylcarnitine, isovalerylcarnitine and methylbutyrylcarnitine (C5), 3-hydroxybutyrylcarnitine (3OH-C4), 3-hydroxyisovalerylcarnitine and 2-methyl-3-hydroxybutyrylcarnitine (3OH-C5), methylmalonylcarnitine (C4DC), hexanoylcarnitine, octanoylcarnitine, malonylcarnitine, decenoylcarnitine, decanoylcarnitine, glutaryl- and 3OH-decanoylcarnitine, dodecanoylcarnitine, methylglutarylcarnitine, 3OH-dodecanoylcarnitine, tetradecadienoylcarnitine, tetradecenoylcarnitine, tetradecanoylcarnitine, suberylcarnitine, 3OH-tetradecenoylcarnitine, 3OH-tetradecanoylcarnitine, hexadecenoylcarnitine, palmitoylcarnitine, sebacylcarnitine, 3OH-hexadecenoylcarnitine, 3OH-palmitoylcarnitine, octadienoylcarnitine, octadecenoylcarnitine, octadecanoylcarnitine, 3OH-octadecenoylcarnitine and 3OH-octadecanoylcarnitine.

The ratios of individual acylcarnitines to C0 were used to determine the host metabolic changes. Two specific ratios representing amino acid metabolism were also calculated: (C5 + 3OHC5)/leucine and citrulline/ornithine.

Faecal total SCFA concentration was calculated as the sum of acetate, propionate, butyrate, valerate, isobutyrate and isovalerate. The proportions of individual SCFA were expressed relative to total SCFA.

Normalization of qPCR data was expressed by all qPCR data relative to the amount of total bacterial measured.

### Statistical analyses

Data were analysed using the Wilcoxon-Mann–Whitney test to compare difference between two groups. For this, four data sets were built: one with all parameters measured in faeces, one with all the ratios calculated from the faecal parameters, one with all metabolites assessed in the blood, and one with all the ratios calculated from the blood. For each data set, the Wilcoxon-Mann–Whitney test was run separately. To correct for false discovery rates, *P* values were adjusted (*q* value) using the Benjamini-Hochberg test. Valerate was only detected in two faecal samples in IBD dogs hence this parameter was not taken into consideration in the statistical analysis. These analyses were done in R (The R Foundation for Statistical Computing, version 3.1.0) using the Coin package (version1.0–23).

Spearman correlations coefficients were calculated between CCECAI and the abundance of different bacterial groups, faecal fermentation end products, plasma amino acids and acylcarnitine profile. The CCECAI score assigned to control dogs was zero (0). Spearman correlation analysis was performed using SPSS version 20 (SPSS Inc., Chicago, Illinois, USA).

For all parameters, statistical significance was accepted at *P* < 0.05 and *q* < 0.05.

## Results

### Study population and CCECAI scores

The IBD group consisted of seven intact females, four spayed females, nine intact males and three neutered males (0.8-12.3 years, median age: 3.8 years). Breeds included Dogue de Bordeaux, Spanish Water Dog, Galgo Español, Golden Retriever, Sheltie, Jack Russell Terrier, Miniature Pinscher, Foxhound, Eurasier, Alaskan Malamute, Bouvier des Flandres, Husky, Great Swiss Mountain Dog, Whippet, Standard Poodle, Lapphund, German Shepherd, Rottweiler, Bichon Frise, Beagle, French Bulldog and Mongrel dogs (×2). Five dogs had insignificant IBD (median CCECAI = 2.0), five dogs had mild IBD (median CCECAI = 5.0), six dogs had moderate IBD (CCECAI = 8.0) and seven dogs had severe IBD (median CCECAI =10).

The control group consisted of five intact females and five intact males (0.5–8.0 years, median age: 2.3 years). Breeds included American Staffordshire Terrier, Staffordshire Bull Terrier, German Shepherd, Scottish Collie, Labrador Retriever, Jack Russell Terrier, Beagle, and Mongrel dogs (×3).

### Faecal microbiota

No differences were found between IBD and control dogs for the relative abundance of Firmicutes, Bacteroidetes, Enterobacteriaceae, *Lactobacillus*, clostridial cluster I, IV and XIVa in faecal samples, nor for the number of gene copies of butyryl-CoA acetate-CoA transferase (BCoAT) and dissimilative sulphate-reducing bacteria gene (dSRB) (*q* > 0.05) (Table 2).

**Table 2:** The faecal abundance of bacterial groups and functional gene in IBD and control dogs. (IBD: *n* = 15, Control: *n* = 10)^a^

	Control		IBD		*q* value
	Mean	SD	Mean	SD	
Absolute value^a^					
Total bacteria	10.49	0.74	10.48	0.59	0.860
Relative value (%)^b^					
Firmicutes	110.6	30.8	127.2	42.1	0.790
Bacteroidetes	35.5	44.6	52.2	51.9	0.823
Enterobacteriaceae	0.6	1.0	3.1	5.5	0.937
*Lactobacillus*	8.7	10.4	1.1	3.5	0.145
Clostridial cluster I	13.1	23.9	13.0	16.3	0.790
Clostridial cluster IV	9.4	9.7	8.5	12.5	0.937
Clostridial cluster XIVa	6.1	5.3	8.4	8.6	0.790
BCoAT	0.090	0.083	0.061	0.071	0.823
dSRB	0.002	0.001	0.001	0.002	0.608

IBD: inflammatory bowel disease; BCoAT: butyryl-CoA acetate-CoA transferase gene; dSRB: dissimilative sulphate-reducing bacteria gene

^a^Results of bacterial groups are expressed as log_10_16S rRNA gene copies/g fresh weight

^b^The relative values are expressed as the ratio of the abundance of bacteria to the abundance of total bacteria

**Fig. 1:**
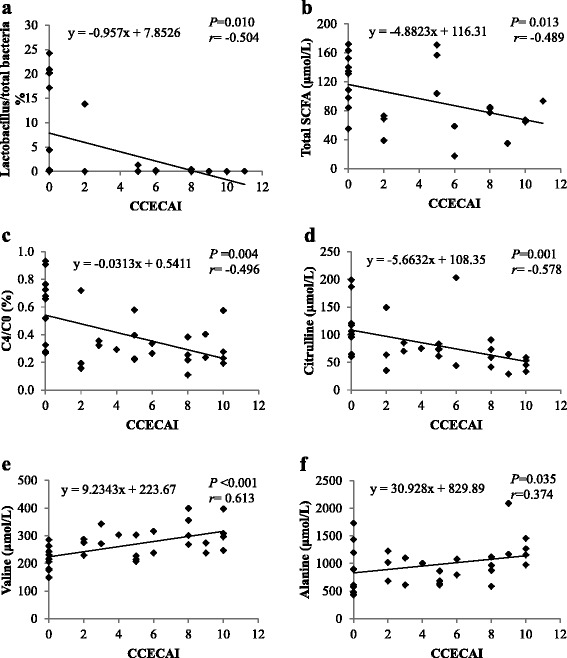
Correlations between canine chronic enteropathy clinical activity index (CCECAI) and several parameters. **a** The relative faecal abundance of *Lactobacillus* (*n* = 25); **b** total faecal SCFA concentrations (*n* = 25); **c** the ratio of plasma C4 (butyrylcarnitine + isobutyrylcarnitine) to C0 (free carnitine) (*n* = 32); **d** plasma valine (*n* = 32); **e** plasma citrulline (*n* = 32); and **f** plasma alanine concentrations (*n* = 32). Figure illustration: The faecal proportions of Lactobacillus are negatively correlated with CCECAI scores **a**; the total SCFA concentrations are negatively correlated with CCECAI scores **b**; the C4/C0 ratios are negatively correlated with CCECAI scores **c**; the plasma citrilline concentrations are negatively correlated with CCECAI scores **d**; the plasma valine concentrations are positively correlated with CCECAI scores **e**; the plasma alanine concentrations are negatively correlated with CCECAI scores **f**

Correlations between CCECAI scores and bacterial groups revealed a negative correlation with relative faecal abundance of *Lactobacillus* (*r* = −0.504, *P* = 0.010) (Fig. 1a).

### Faecal fermentation metabolites

No differences were observed between IBD and control dogs for faecal ammonia and SCFA concentrations (*q* > 0.05; Table 3). Spearman’s correlation showed only a negative correlation between faecal total SCFA concentrations and CCECAI scores (*r* = −0.489, *P* = 0.013) (Fig. 1b).

**Table 3:** Faecal concentrations of fermentation end-products in IBD and control dogs. (IBD: *n* = 15, Control: *n* = 10)^a^

	Control		IBD		*q* value
	Mean	SD	Mean	SD	
Acetate (%)	63.0	11.6	61.9	11.8	0.937
Propionate (%)	26.6	10.5	24.8	12.0	0.937
Butyrate (%)	7.0	3.7	10.1	4.4	0.726
Isobutyrate (%)	1.5	1.4	0.8	1.0	0.608
Isovalerate (%)	1.8	2.4	1.5	1.7	0.937
Ammonia (μmol/g)	324	195	238	86	0.843
Total SCFA (μmol/g)	123.9	36.9	80.1	39.7	0.145

IBD: inflammatory bowel disease; SCFA: short-chain fatty acids

^a^The relative values are expressed as the ratio of individual SCFA to total SCFA

### Blood parameters

Serum cobalamin concentrations did not differ between the IBD and control group, while serum folate concentrations were lower in the IBD compared to the control group (*q* = 0.023) (Table 4). The IBD group also showed increased plasma valine concentrations (*q =* 0.003), whereas plasma citrulline concentrations (*q* = 0.016) and citrulline/ornithine ratios (*q =* 0.014) were decreased in IBD compared to control dogs (Table 4). Also plasma C0 (*q =* 0.003) and total acylcarnitine (*q =* 0.003) concentrations were increased in IBD compared to control dogs (Table 4). As for the ratios of individual plasma acylcarnitines to C0, C4/C0 (*q =* 0.008) and C4DC/C0 (*q =* 0.006) were decreased, while (C5 + 3OHC5)/leucine (*q =* 0.006) were increased in IBD compared to control group (Table 4). For C3/C0 and 3OHC4/C0 no significant difference was observed between groups (Table 4).

**Table 4:** Blood parameters of IBD and control dogs. (IBD: *n* = 15, Control: *n* = 10)

	Control		IBD		*q* value
	Mean	SD	Mean	SD	
Cobalamin (pmol/L)^a^	285	68.1	301	213	0.651
Folate (pmol/L)^b^	34.5	6.75	24.0	11.5	0.023
Indoxyl sulfate (mg/dL)	1.56	1.05	1.57	1.65	0.681
Plasma amino acids					
Valine (μmol/L)	213	43.1	287	53.8	0.003
Leucine (μmol/L)	257	58.3	274	61.0	0.536
Ornithine (μmol/L)	32.6	7.42	37.7	8.41	0.187
Citrulline (μmol/L)	112	48.1	71.3	39.0	0.016
Citrulline/Ornithine	3.53	1.77	2.00	1.07	0.014
Plasma acylcarnitine profile					
C0 (μmol/l)	16.2	13.7	36.1	21.9	0.003
Total acylcarnitines (μmol/L)	2.16	1.03	4.00	1.84	0.003
Total acylcarnitines/C0 (%)	16.4	6.9	12.4	4.7	0.113
C2/C0 (%)	11.0	4.8	7.6	3.6	0.096
C3/C0 (%)	0.587	0.483	0.749	0.796	0.643
C4/C0 (%)	0.606	0.248	0.316	0.149	0.008
C4DC/C0 (%)	0.374	0.172	0.179	0.103	0.006
3OHC4/C0 (%)	0.111	0.083	0.119	0.078	0.874
(C5 + 3OHC5)/Leucine (%)	0.034	0.028	0.060	0.024	0.006

IBD: inflammatory bowel disease; C0: free carnitine, C2: acetylcarnitine, C3: propionylcarnitine, C4: butyrylcarnitine + isobutyrylcarnitine, C4DC: methylmalonylcarnitine, 3OHC4: 3-hydroxybutyrylcarnitine, C5: isovalerylcarnitine + methylbutyrylcarnitine, 3OHC5: 3-hydroxyisovalerylcarnitine + 2-methyl-3-hydroxybutyrylcarnitine

^a^ Reference range of cobalamin: 73–485 pmol/L. One dog in IBD group is out of reference range

^b^ Reference range of folate: 9.0–36.0 nmol/L. Five dogs in IBD group and four dogs in control group are out of reference range

Negative correlations between CCECAI scores and C4/C0 (*r* = −0.496, *P =* 0.004), and between CCECAI scores and plasma citrulline concentrations (*r* = −0.578, *P =* 0.001) were observed (Fig. 1c and d). In addition, positive correlations were noted between CCECAI scores and plasma valine (*r* = 0.613, *P* < 0.001), and between CCECAI scores and plasma alanine (*r* = 0.374, *P =* 0.035) concentrations (Fig. 1e and f).

## Discussion

This study aimed to provide new insights into the relationship between faecal microbiota profile and their functionality, and host metabolic changes in canine IBD. Although changes in gut microbiota, and in particular the numbers of *Faecalibacterium* spp. have been repeatedly reported to be decreased in IBD dogs [4, 7], no major changes in faecal microbiota have been observed in this study. In addition, butyrate producing bacteria were also not affected by the disease status in the present study. The fact that no significant differences were observed in faecal microbiota between IBD and control dogs could be due to three main reasons: (1) A large inter-individual variability among dogs may exist and has been observed in other canine studies [27, 28]. The sources of the variation may include gender, age, breed, living conditions (e.g. exposure to pathogens, people, or other animals), diet (e.g. macronutrients composition or form) and disease activity. To search for breed and age matched IBD and healthy dogs was the initial goal of the study but was impossible to put into practice. More standardized requirements (e.g. diet and living condition) might reduce the large inter-individual variability among dogs. (2) The methodology that was used (i.e. DNA extraction and qPCR). Although qPCR is the most accurate culture-independent measure of the microbial abundance [29], 16S rRNA sequencing and metagenomic sequencing could provide a more in-depth characterisation of overall faecal microbiota shifts, whereas the latter can determine the microbiome in terms of potential functions.(3) Sample types. Most studies using duodenal biopsies have detected distinct differences in gut microbial community between IBD and healthy dogs [5, 6]. Although faecal samples are non-invasive to obtain, it remains a debate if faecal microbiota data appropriate to study gut microbiota accurately [30, 31].

Nevertheless, the relative abundance of *Lactobacillus* gradually decreased with increased severity of IBD in dogs (Fig. 1). A reduction in faecal *Lactobacillus* has been previously reported in human IBD patients [32]. Several *Lactobacillus* strains have been proven to effectively protect against IBD in murine models through down-regulation of pro-inflammatory cytokines [33, 34]. Although no difference in abundance of *Lactobacillus* has been observed previously [3], the probiotic VSL#3 containing several strains of *Lactobacillus* and other bacterial strains showed a potential protective effect in IBD dogs [4]. Therefore, more in-depth studies are needed to identify the *Lactobacillus* strains that are altered in canine IBD.

Folate absorption occurs in the jejunum and abnormalities of the proximal small intestinal mucosa may lead to reduced serum folate concentrations [35], as seen in IBD dogs included in the present study. In addition, gut commensals including *Bifidobacteria* and *Lactobacillus* can produce folate [36]. Therefore, decreased serum folate concentrations may be a consequence of impaired intestinal function and/or altered gut microbial community (e.g. the observed decrease in the abundance of *Lactobacillus*). Whether alteration in *Lactobacillus* contributes to a decrease in serum folate needs further investigation.

In this study, IBD dogs were characterized by distinctive metabolic profile with changes in plasma free amino acids and acylcarnitines. This could be due to two possible reasons: (1) tissue damage, and (2) host metabolic changes. Eighteen out of 23 IBD dogs experienced different magnitude of weight loss (see Additional file 1), which suggested the occurrence of tissue loss, e.g. the adipose tissue and lean mass. Extreme weight loss with muscle wasting (known as “cachexia”) may occur in dogs with severe IBD [37]. In addition, intestinal mass might be reduced as citrulline mainly originates from duodenum and jejunum, and its blood level is highly dependent on small bowel enterocytes mass in humans [38]. However, inter-species difference on citrulline conversion may exist [39]. Free carnitine and acylcarnitine concentrations in tissues (muscle, intestine, etc.) are higher than in the blood [40–42]. Thus, tissue loss might lead to an increase in plasma C0 and total acylcarnitine concentrations in IBD dogs. On the other hand, these results might suggest an alteration on host metabolism. Use of fat stores for ketone production and direct oxidation of fat as a primary fuel are characteristic of starvation. Previous study reported increased ketone body in dogs with IBD, which suggested an energy insufficiency [3]. Additionally, prolonged fasting was associated with increased plasma and muscle free carnitine and total carnitine in dogs [43]. Thus, the use of amino acids and fatty acids as energy source might be expected in dogs with IBD.

Moreover, IBD dogs showed reduced plasma C4/C0 and this decrease was associated with the severity of IBD. This could indicate a drop in butyrate and/or isobutyrate metabolism in liver [18, 44]. Additionally, the ratio of C4DC, which originates from valine and propionate [44], to C0 was also declined in IBD dogs. The drop of C4DC was more likely due to a lack of propionate because plasma valine was significantly increased. These results are in accordance with previous studies in humans that demonstrated short-chain acylcarnitines, including C3, C4 and C5, to be significantly lower in CD and UC patients compared to healthy controls [45, 46]. The decreases of these short-chain acylcarnitines are supported by decreased SCFA in more severe IBD dogs. Although faecal SCFA did not differ between IBD and control dogs, this could be due to rapid absorption of SCFA leaving only a small detectable amount in faeces. It is not known if the SCFA derived from intestinal fermentation are a substantial source of energy in dogs, but the shifts at least point to a change in nutrient metabolism that is associated with the events in the intestinal lumen.

Inflammatory conditions are known to stimulate protein catabolism and release amino acids from muscle tissue to provide substrate for proteins of the immune system [19]. Alanine is an important transport metabolite for amino groups in animals, therefore, increased concentrations of alanine might indicate a higher amino acid turnover [44, 47]. In addition, increased plasma valine concentrations and (C5 + 3OHC5)/leucine ratios might be associated with the increase in branched-chain amino acids catabolism in the host [43]. However, a decrease in serum alanine has been observed in dextran sulfate sodium induced UC mice [19] and decreases in serum valine have been reported in both UC and CD human patients [20]. The reason for the different observations between dogs and other species is not known. However, alteration in plasma amino acids concentration could also be involved with increased mobilisation from lean tissues. In contrast, a decrease in plasma citrulline/ornitine ratio suggested decreased protein catabolism. This contradicted with the previous suggestion of increased degradation. As mentioned previously, citrulline is an amino acid released exclusively from small bowel enterocytes, a disease condition involving the upper intestine could reduce its production in humans [48]. Notably, an increased citrulline level was observed in dogs with IBD that received probiotic treatment compared to untreated dogs [4]. Therefore, future studies are warranted to evaluate if plasma citrulline could be a putative marker of intestinal function in canine IBD.

## Conclusions

Canine IBD is characterised by significant changes in the nutritional metabolic profile especially in the SCFA and amino acids metabolism. A decrease in citrulline is highlighted in this study and warrants for future studies to test if it could be a putative marker of intestinal function in canine IBD. However, in contrast to previous studies, no major changes in the bacterial groups were observed in the present study. This could be mainly due to the large inter-individual variability and the technique used. Nevertheless, dogs with more severe of IBD were associated with decreased faecal proportion of *Lactobacillus.*

## Abbreviations

3OH-C4, 3-hydroxybutyrylcarnitine; 3OH-C5, 3-hydroxyisovalerylcarnitine and 2-methyl-3-hydroxybutyrylcarnitine; BCoAT, butyryl-CoA acetate-CoA transferase; C0, free carnitine; C2, acetylcarnitine; C3, propionylcarnitine (C3); C4, butyrylcarnitine and isobutyrylcarnitine; C4DC, methylmalonylcarnitine; C5, tiglylcarnitine, isovalerylcarnitine and methylbutyrylcarnitine; CCECAI, canine chronic entreropathy clinical activity index; CoA, co-enzyme A; dSRB, dissimilative sulphate-reducing bacteria gene; IBD, inflammatory bowel disease; SCFA, short-chain fatty acids; UC, ulcerative colitis.

## Additional file

Additional file 1: Canine chronic entreropathy clinical activity index in IBD dogs. Description of data: the total CCECAI score and the individual score of each criterion. (XLSX 9 kb)
